# Treatment of periodontal diseases: Latin America and the Caribbean Consensus 2024

**DOI:** 10.1590/1807-3107bor-2024.vol38.0121

**Published:** 2024-11-22

**Authors:** Ricardo Guimarães FISCHER, Guilherme Castro Lima Silva do AMARAL, Aldrin André HUAMÁN-MENDOZA, Luis Rossy BUENO, Cristina Cunha VILLAR

**Affiliations:** (a) Pontifícia Universidade Católica do Rio de Janeiro – PUCRJ, School of Dentistry, Department of Periodontics, Rio de Janeiro, RJ, Brazil.; (b) Universidade de São Paulo – USP, School of Dentistry, Department of Periodontics, São Paulo, SP, Brazil.; (c) Fundación Juan José Carraro, Buenos Aires, Argentina.

**Keywords:** Periodontal Diseases, Dental Care, Public Health, Latin America, Caribbean Region

## Abstract

The prevalence of periodontitis in Latin American and Caribbean countries (LACC) drew attention to a significant public health issue exacerbated by socio-economic disparities. This consensus report, based on the European Federation of Periodontology (EFP) S3 level clinical practice guidelines, proposed a multifaceted approach to periodontal health-care. The report highlighted the critical need for holistic, population-wide health policies and underscored the lack of documented community interventions in contemporary literature. The consensus advocated for a patient-centered approach to periodontal care, with non-surgical and surgical interventions, and a long-term commitment to supportive periodontal care (SPC). It emphasized the importance of patient engagement in biofilm control by means of home-care and professional interventions for long-term periodontal health. The report also stressed that subgingival instrumentation benefits even severely compromised teeth, by significantly reducing probing depths and gingival inflammation. Moreover, it emphasized the importance of personalized, long-term SPC for maintaining oral health post-treatment, and pointed out the need to identify factors influencing patient adherence. The aim of this report was to provide actionable guidance for clinicians and policymakers, focused on improving periodontal health outcomes and quality of life in LACC.

## Introduction

The dynamic and diverse panorama of periodontal healthcare in Latin America and the Caribbean countries (LACC) reflects this region’s multifaceted cultural and geographical mosaic. Within this context, periodontal disease emerges as a substantial health concern, necessitating a comprehensive approach that encompasses all aspects of periodontal treatment, from the initial stages of oral hygiene instructions (OHI) to the pivotal phase of supportive periodontal care (SPC). Building upon this framework, this report synthesizes contemporary scientific knowledge and the EFP S3 level clinical practice guideline^1^ to support clinical decisions and shape cost-effective public policies. We explored the full spectrum of periodontal therapy, addressing the initial phase of risk factor control, non-surgical subgingival instrumentation, subsequent reinterventions or surgical interventions when necessary, and SPC for patients with Stages I-III periodontitis. The treatment approach to periodontitis is stage-dependent, requiring a pre-established, gradual sequence of interventions, with more complex and costly procedures as the disease progresses. Our focus is to guide practitioners and policymakers toward evidence-based treatments, emphasizing the role of Primary Health Care and addressing the unique challenges within this region. The aim of this review is to provide insights into, and practical guidelines to enhance periodontal health and quality of life across LACC.

### Periodontal treatment: first step

The first phase of periodontal therapy is crucial for motivating patients to change behaviors, especially in effectively removing supragingival biofilm and managing risk factors for periodontal disease, and this is applicable to all stages and grades of periodontitis.^1^

#### Home-care treatment

Effective control of supragingival biofilm hinges on guiding patients towards improved oral hygiene and behavioral changes.^1^ Brushing twice daily for at least two minutes is essential, although the best brushing technique and duration are still under debate.^2,3^An 11-year study demonstrated that brushing twice daily significantly reduced the number of teeth with probing depths (PD) ≥ 4 mm.^4^ However, minimizing excessive brushing force is important to avoid gingival recession and non-carious cervical lesions (NCCLs).^3^ While powered toothbrushes may enhance patient compliance,^5^ two studies from Brazil found no significant differences among ultrasonic, electric, and manual brushes in clinical and microbiological outcomes.^6,7^ However, systematic reviews have indicated that powered toothbrushes are generally more effective in reducing gingivitis and biofilm,^8,9^ leading to an additional 11% reduction in gingivitis and an additional 21% reduction in supragingival biofilm. Interdental brushes are preferred for interproximal cleaning, as they significantly reduced gingival inflammation.^10,1^ According to a Brazilian study, patients who did not perform interproximal cleaning are 2.19 times more likely to develop gingivitis.^11^ Psychological interventions such as cognitive behavioral therapy and motivational interviewing have shown limited effectiveness in improving oral hygiene habits.^12,1^

#### Professional treatment

Professional supragingival biofilm removal (PSBR) and management of factors retentive to biofilm are essential for primary and secondary prevention of periodontal diseases.^1^ A split-mouth clinical trial in Brazil revealed that PSBR reduced the need for subgingival procedures by 48%.^13^PSBR also helps maintain periodontal stability during SPC.^14^

#### Risk factor control

Effective risk management, especially targeting tobacco smoking and diabetes, is crucial for periodontal health.^1^ Smoking cessation strategies such as the ‘5 A’s’ model and ‘5 R’s’ approach are effective.^15^ Economic analysis in Brazil has shown the cost-effectiveness of smoking cessation programs for periodontitis patients, emphasizing their role in preventing tooth loss and enhancing quality of life.^16^ A two-year longitudinal study in Brazil indicated that smoking cessation led to gains in clinical attachment level (CAL) and reduced PD.^17^A systematic review of longitudinal studies revealed that the risk of tooth loss for former smokers was similar to that for non-smokers (Relative Risk [RR]=1.15, 95% CI=0.98-1.35), in contrast to current smokers who faced a significantly higher risk (RR=2.60, 95% CI=2.29-2.96).^18^The length of smoking cessation is key in mitigating risks.^18,19^ Successful smoking cessation predictors in Brazilian periodontitis patients included being male, not living with smokers, and showing low nicotine dependence.^20^

Diabetes management is also crucial for enhancing periodontal treatment outcomes and ensuring long-term stability in periodontitis patients.^21^ Educational interventions, dietary counseling, and referrals for blood glucose management are essential.^1,21^ While no direct evidence has linked physical activity and weight loss to periodontal outcomes, these lifestyle changes may indirectly benefit periodontal health by reducing inflammation, improving bone density, and increasing insulin.^22^

## Periodontal treatment: second step

The second stage of periodontal treatment emphasizes the removal of calculus and subgingival biofilm by means of meticulous subgingival instrumentation.^1,23^ This technique is effective even for severely compromised teeth and aims to reduce PD, gingival inflammation, and the number of diseased sites.^24-27^ Nevertheless, the success of this stage depends heavily on successful implementation of the first step of periodontal treatment.^1^ Subgingival instrumentation results in a reduction of 2.2 mm in PD, and a 0.5–2 mm gain in CAL in deep sites.^24-26^ A recent meta-analysis reported an increase from 39.1% to 64.1% in sites with PD < 3mm after subgingival instrumentation, reflecting a significant rise in the number of healthier sites.^28^Furthermore, the meta-analysis revealed a significant reduction of 15.5% in sites with PD ≥ 5 mm (95%CI: 7.86–23.13, p < 0.001), with the mean percentage of sites with PD ≥ 5 mm decreasing from 28.23% to 11.71% before and after treatment. Notably, four studies included in this meta-analysis were conducted in Latin America: three from Brazil^29-31^ and one from Chile.^32^However, the treatment efficacy varies depending on factors such as tooth type, with non-molars showing better response than molars, extent of periodontal destruction, local factors, and patient age.^33^ For instance, while 75% of all pockets resolved in patients with stage II periodontitis, the closure rates were approximately 66% and 50% in localized and generalized stage III–IV periodontitis, respectively.^28^ Furthermore, this shows that if non-surgical subgingival instrumentation were the only therapy applied in severe periodontitis, it may be ineffective in achieving periodontal stability over time.

Contemporary guidelines do not specify the number of sessions for subgingival instrumentation but caution against potential systemic risks with full-mouth disinfection.^1^ Both hand instruments and sonic/ultrasonic devices, used individually or in combination, are recommended for effective subgingival instrumentation.^34^

Despite potential benefits, including in patients with diabetes^35^ - the European Federation of Periodontology (EFP) advises against the combined use of lasers and antimicrobial photodynamic therapy (aPDT) with subgingival instrumentation due to limited supporting evidence.^36^ Similarly, the adjunctive use of sustained-release local antimicrobials and host-modulating agents such as statins, probiotics, sub-antimicrobial doxycycline, bisphosphonates, non-steroidal anti-inflammatory drugs (NSAIDs), omega-3 polyunsaturated fatty acids, metformin, doxycycline, tetracycline, and minocycline have demonstrated some clinical benefits. However, their use is not recommended due to limited evidence, potential biases in the studies, lack of standard protocols, and potential side effects.^1,37-39^

As regards antiseptic mouthwashes, a systematic review pointed out that the use of chlorhexidine-based mouthwash adjuvant to subgingival instrumentation can significantly reduce PD at 40 and 60 days (Mean Difference [MD] = 0.33 mm, 95%CI: 0.08–0.58, p = 0.01) and at 180 days (MD = 0.24mm, 95%CI: 0.02–0.47, p = 0.035), without affecting CAL when compared with subgingival instrumentation alone.^40^ It is noteworthy that four Brazilian randomized clinical trials were included in this systematic review and meta-analysis.^29,41-43^ Moreover, studies on the Brazilian population have reported that the adjunctive use of chlorhexidine mouthwash improves not only clinical but also microbiological parameters, by reducing the levels of red and orange complexes and increasing the presence of symbiotic species.^41-43^ Nonetheless, its use should only be considered in patients with adequate plaque control, taking into account potential side effects and costs.^1^

When considering antibiotics as adjunctive therapy, systemic antibiotics are particularly effective in young patients with generalized Stage III or IV periodontitis.^1^ A recent systematic review concluded that systemic antibiotics, especially a combination of adjunct metronidazole (MTZ) and amoxicillin (AMX), significantly reduced the number of sites with PD exceeding 5 mm by 40% to 50% and significantly improved CAL.^44^ Notably, 11 out of the 28 studies included in this review were conducted in Latin America: nine in Brazil, one in Chile, and one in Colombia.^44^ Although there is a body of strong evidence supporting the additional effects of systemic antibiotics, their routine adjunctive use in periodontal treatment is discouraged due to health risks and concerns about antibiotic resistance.^44,45^

In summary, while subgingival instrumentation continues to be pivotal in periodontal therapy, the efficacy of adjunct methods requires thorough evaluation considering the associated risks, benefits, and quality of evidence. Further research is needed to establish clear guidelines and protocols for their use in LAAC.

## Periodontal treatment: third step

After the second step of periodontal treatment, a periodontal re-evaluation is required to assess the individual´s healing response. Since maintaining teeth with adequate health, function, and esthetics is challenging to report and requires long periods of evaluation, surrogate measures such as changes in PD, CAL and inflammation indices (e.g. reduction in bleeding of probing) are used.^46^ The proposed endpoints for successful treatment include the absence of PD ≥ 4 mm with bleeding on probing (BOP) and no PD ≥ 6 mm. However, these endpoints are often not achieved. In such cases, a third step of therapy must be implemented. This may include repeated subgingival instrumentation with or without adjunctive therapies, access flap surgery (AFS) for improved access, and resective and regenerative surgeries to reduce periodontal defects, particularly furcation and vertical defects.^1^

When comparing AFS with non-surgical subgingival instrumentation, the effectiveness depends on the initial PD. AFS has been demonstrated to show significantly greater reduction in PS in initially deep pockets (PD ≥ 6 mm) in both short-term (< 1 year, MD = 0.67 mm, 95%CI: 0.37–0.97) and long-term studies (≥ 1year, MD = 0.39 mm, 95%CI: 0.09–0.70), without significant differences in CAL gain and patient preference.^47^ However, in shallow pockets, AFS has resulted in significantly greater CAL loss in both short-term (MD = 0.43 mm, 95%CI: 0.56,-0.29) and long-term evaluations (MD = -0.27 mm, 95%CI:-0.34,-0.20).^47^ Notably, this systematic review included a Brazilian randomized clinical trial that showed both minimally invasive surgical and non-surgical approaches led to significant reductions in PD and gains in CAL, with no significant differences between the two groups.^48^ Similar studies have demonstrated that the percentage of residual sites with PD > 3 mm after treatment varied from 17% to 49% after AFS, and 20% to 62% after subgingival instrumentation.^49-53^

A systematic review and meta-analysis demonstrated that pocket reduction/elimination techniques were superior to AFS 6–12 months post-surgery (MD = 0.47 mm, 95%CI: 0.7–0.24), especially in sites with an initial PD ≥ 6 mm. However, longer-term follow-up (36–60 months) did not reveal significant differences between the two surgical approaches.^54^

As regards regenerative surgeries for the treatment of vertical defects, all regenerative therapies resulted in better clinical outcomes when compared with AFS. Both guided tissue regeneration (GTR) and enamel matrix derivative (EMD) significantly enhanced CAL gain in intrabony defects compared with AFS alone (MD = 1.43 mm, 95%CI: 0.76–2.22; and MD = 1.27 mm; 95%CI=0.79–1.74, respectively).^55^ In a recent Brazilian randomized clinical trial involving patients with controlled type 2 diabetes mellitus, the treatment of vertical defects using a simplified papilla preservation flap with and without EMD were compared. The defects treated with papilla preservation flap and EMD exhibited significantly greater CAL gain (3.31 ± 0.96 mm vs 1.61 ± 1.12 mm, p = 0.001) and PD reduction (5.15 ± 1.21 mm vs 2.84 ± 0.98 mm, p = 0.001) compared with the defects treated with the papilla preservation flap alone, at 6 months follow-up.^56^ Furthermore, periodontal regeneration using EMD or bone grafts with or without resorbable membranes is also indicated for mandibular or maxillary buccal class II furcations.^1^

## Costs

An important consideration in periodontal therapy is the extra cost of surgery, which adds 746 Euros per patient to the costs over 6 months in comparison with subgingival instrumentation alone. However, at 12 months, 46 Euros of this cost could be offset due to a reduced need for SPC and systemic antibiotics.^57^ A study by The Economist found that professionally managed periodontitis is cost-effective in European countries. Unfortunately, comparable data for LACC is lacking. Differences in healthcare systems, economic conditions, and patient demographics mean that findings from other regions may not apply directly to LACC. Therefore, further research specifically related to LACC countries is necessary to understand the economic and clinical implications of periodontal therapy options in these diverse healthcare environments.

In LACC, a key challenge is the cost barrier to accessing dental services, particularly for low-income families. Dental care is often primarily available through public services, universities, and military dental services, but these may be limited in scope and reach. In Brazil, specialized public-health clinics known as Dental Specialties Centers (DSC) provide periodontal surgeries after a referral from the Family Health Strategy (FHS).^58^ A major difficulty with this system is inadequate periodontal diagnosis at the FHS level since this leads to overbooking at DSCs.^59^ Furthermore, the appointment control center lacks protocols for prioritizing care, and there is a scarcity of DSCs throughout the country. Public coverage of dental care for periodontitis needs to be reviewed by policymakers and commissioners across LACC to ensure equitable access to necessary treatments and improve overall public health outcomes in the region

## Supportive periodontal care

Both LACC dentists and patients need to understand the significance of SPC, as it is a key procedure in preventing the recurrence of periodontal disease and in promoting long-term oral health after periodontal therapy. This involves updating medical and dental histories, managing risk factors such as smoking and diabetes, and promoting behavioral changes that include good oral hygiene and adherence to maintenance schedules.^1^ During clinical examinations, periodontal and peri-implant conditions are assessed, and this allows for tailored OHI. SPC also includes removing factors that promote plaque-retention and supragingival biofilm, polishing, and subgingival instrumentation of moderate and deep sites. A Brazilian study pointed out that oral prophylaxis, combined with OHI and subgingival instrumentation were more effective in reducing probing depths ≥ 5 mm than OHI and prophylaxis alone during SPC.^60^

### Home-care therapy during SPC

In specific cases, antiseptic mouthwashes and dentifrices are recommended to control gingivitis during SPC. Mouthrinse options include those with essential oils, chlorhexidine, and cetylpyridinium chloride. For dentifrices, formulations with triclosan-copolymer, chlorhexidine, and stannous fluoride-sodium hexametaphosphate are considered effective.^1^ A Brazilian randomized controlled trial with a 2-year follow-up demonstrated that dentifrice containing 0.3% triclosan + 2.0% PVM/MA copolymer was more effective than regular fluoride dentifrice in reducing BOP, plaque index, and the percentage of sites with PD greater than 4 mm during the SPC.^61^

## Determining SPC Frequency

The ideal frequency for SPC is subject to debate, with recommended intervals ranging from two weeks to 18 months. Longitudinal studies with the aim of tailoring SPC frequency to individual risk profiles have yielded mixed results. For example, Matuliene et al.^62^ categorized 160 patients into risk categories, suggesting annual sessions for low-risk patients and up to four sessions yearly for high-risk patients. Despite increased SPC frequency, higher risk was associated with more tooth loss. Similarly, Trombelli et al.^63^ observed varying tooth loss rates across risk groups despite their comparable SPC schedules. A Brazilian study^64^ found monthly visits improved plaque scores but did not significantly alter other periodontal measures when compared with three-month intervals. Recent research by Ravidà et al.^65^ suggested SPC visit frequencies based on periodontitis severity: every 7.4 months for stages I-II, 6.7 months for III-IV, 7.2 months for grade B, and 6.7 months for grade C, with shorter intervals recommended for smokers, diabetics, and the elderly.

## Adherence to SPC

Adherence to SPC is vital to prevent tooth loss and recurrent periodontitis. Non-adherence leads to a 26% higher risk of tooth loss^66^ and an increased risk of periodontitis progression.^67^ Regular SPC adherence in Brazil significantly reduced annual tooth loss from 0.36 to 0.12 teeth/year.^68^ Adherence rates vary widely, ranging from 11% to 88%. A Brazilian study indicated only 26% of patients consistently returned for SPC, with 40% doing so irregularly.^69^ Discontinuation of SPC is more common in the first few years.^70^ Factors influencing discontinuation include age, female gender, personality traits such as anxiety, dental fear, systemic health conditions, smoking, socio-economic status, and lack of information.^70,71^ A Brazilian study noted women under 30 or over 51, particularly those undergoing non-surgical therapy, were more likely to be non-compliant.^72^ However, factors such as smoking cessation, older age, low percentage of BOP, severe periodontal disease, longer active treatment duration, and extended SPC intervals improve adherence.^71^ Regional differences, across Brazil, Venezuela, Chile, and Argentina, emphasized the impact of cultural and socio-economic conditions, and oral hygiene knowledge on SPC adherence,^73^ highlighting the need for tailored approaches to SPC adherence strategies.

### Long-term periodontal outcomes during SPC

The average annual tooth loss among SPC patients ranges from 0.1 to 0.2 teeth, with significant patient-specific variations.^74^ A small group of SPC patients was responsible for the majority of tooth loss, which was influenced by factors such as age, gender, smoking, diabetes, advanced periodontitis, and adherence to SPC, as well as specific tooth characteristics such as maxillary and molar teeth, initial PD, number of sites with PD ≥5 mm and involvement of furcation.^65,74,75^ In Brazil, predictors of molar loss during SPC include gingival bleeding, advanced furcation lesions, and patient characteristics such as age over 50, male gender, diabetes, smoking, and non-compliance.^76^ In a 30-year longitudinal study of SPC after periodontal therapy, only 201 teeth (5.1%) were lost, with 39 occurring for periodontal reasons. Periodontitis stages III or IV were associated with greater tooth loss during SPC compared with stages I or II (OR = 2.10; p = 0.048). Patients with generalized periodontitis also showed a statistically significant increase in tooth loss compared with those with localized periodontitis (OR = 3.24; p = 0.016).^77^ Of interest, strict adherence to SPC can mitigate the negative effects of not achieving stable periodontal health after treatment.^78^

A correlation has also been observed between the duration of SPC follow-up and clinical attachment loss. Patients with follow-ups longer than 10 years exhibited a slightly higher incidence of attachment loss (26.3%) compared to those with 5 to 10 years of SPC (22.1%).^79^ This emphasizes the progressive nature of periodontal disease over time and the importance of long-term maintenance. Brazilian studies have linked tooth loss and recurrence of periodontitis during SPC to the male gender, periodontitis severity, surgical therapy, and lifestyle factors such as irregular SPC adherence, poorly controlled diabetes, smoking, intense alcohol use, poor oral hygiene, and depressive disorders .^68,80-84^

Compliance with SPC is crucial in preventing tooth loss, however, it may not be cost-effective for all patients. Compliant patients in more advanced stages of periodontitis (Stage III/IV and Grade B/C) incur lower cumulative costs for relapse treatments.^85^ Conversely, patients diagnosed with stage I/II, grade A periodontitis might benefit financially from fewer SPC visits, with a minimum of one visit per year.^85^ Moreover, there has been a disparity in periodontitis progression and tooth loss between private and public academic patients in Brazil, with lower rates in private settings.^86^ These findings emphasize the complexity of periodontal disease progression and the necessity for tailored, comprehensive SPC strategies that consider both periodontal status, systemic health, and socio-economic factors.

## Social perspectives and challenges of treating periodontitis in LACC

In LACC, the management of periodontitis is inextricably linked to the region’s complex socio-economic landscape. Despite modest regional Gross Domestic Product (GDP) growth, averaging around 2% (World Bank, 2023), the region grapples with extreme poverty and income inequalities, which profoundly affect public health initiatives, including the management of periodontal diseases. Stark income inequality, where the wealthiest 10% of the population earns 55% of total income, while the poorest 50% earns just 10% (CAF, Banco de Desarrollo de América Latina), intensifies these disparities in access to healthcare. Consequently, periodontal disease do not only represent a public health challenge but also serve as indicators of deeper socio-economic inequalities, with a notably higher prevalence in lower socioeconomic groups. Healthcare spending in LACC, at approximately 6.9% of GDP in 2019, is below the OECD (Organization for Economic Co-operation and Development) countries average of 8.5%, and the allocation for dental care is even more constrained. This limited budget fails to address the needs of the regional population, particularly those in lower-income brackets, where the burden of periodontal diseases is most significant. Thus, addressing periodontitis in these regions calls for interventions that are both cost-effective and accessible, focusing on preventive strategies and early diagnosis and interventions.

Addressing periodontitis in LACC also requires a paradigm shift in dental academic institutions, clinical practices, and national dental associations toward adopting evidence-based, feasible, and cost-effective strategies. This shift involves focusing not only on isolated treatment options, but also on structured preventive programs that promote healthy lifestyles. These programs are likely to be the most cost-effective method for optimal periodontal care. Such a transformative approach necessitates ongoing education and regular updates in clinical training to accurately reflect the unique realities of the region. Moreover, the diversity of oral healthcare systems across LACC, influenced by varied economic and political factors, poses challenges to the uniform implementation of these strategies. While many LACC have established national oral health policies focusing on the prevention, diagnosis, treatment, and maintenance of periodontal diseases (as detailed in Table), the effectiveness of these policies in real-world practice remains largely unexplored. The management of periodontitis should follow clinical protocols that are not only tailored to local social and oral health conditions but also to resource availability. These strategies must be both clinically effective and economically feasible, with the goal of ensuring equitable access to oral health services.

**Table:** Oral health policies with periodontal treatment strategies implemented in LACC countries.

Country	Access link
Argentina	https://www.sssalud.gob.ar/pmo/res_s_02_201.pdf
Bolívia	https://www.minsalud.gob.bo/images/Descarga/saludOral/2010-Normas_Salud_Oral-6316.pdf
Brazil	https://aps.saude.gov.br/noticia/22036
Chile	https://www.minsal.cl/wp-content/uploads/2022/02/PLAN-NACIONAL-DE-SALUD-BUCAL-2021-2030.pdf
Costa Rica	https://www.ministeriodesalud.go.cr/index.php/biblioteca-de-archivos-left/documentos-ministerio-de-salud/
Costa Rica	ministerio-de-salud/planes-y-politicas-institucionales/politicas-en-salud-1/5753- politica-nacional-de-salud-bucal-2022-2032/
Ecuador	https://www.salud.gob.ec/wp-content/uploads/2016/09/Protocolos-Odontol%C3%B3gicos.pdf
El Salvador	https://www.transparencia.gob.sv
Honduras	https://secretariadesaludhn.wordpress.com/programas-de-la-secretaria-de-salud/
Mexico	https://minsa.gob.pa/programa/programa-de-salud-bucal
Panamá	https://minsa.gob.pa/programa/programa-de-salud-bucal
Paraguay	https://www.gub.uy/ministerio-salud-publica/comunicacion/publicaciones/programa-nacional-de-salud-bucal
Peru	https://cdn.www.gob.pe/uploads/document/file/306236/Resoluci%C3%B3n_Ministerial_N__324-2019-MINSA.PDF
Dominican Republic	https://sns.gob.do/cartera-servicios-niveles-atencion/
Uruguay	https://www.gub.uy/ministerio-salud-publica/comunicacion/publicaciones/programa-nacional-de-salud-bucal
Venezuela	https://www.sld.cu/galerias/pdf/uvs/saludbucal/presenvenez.pdf

## Conclusions, research gaps, and future needs

### Conclusions

- **Holistic approach:** The consensus emphasized a comprehensive approach to periodontal healthcare, integrating individual risk factor management with a combination of non-surgical and surgical treatments, and a long-term commitment to SPC (Figure).
  **Figure:**
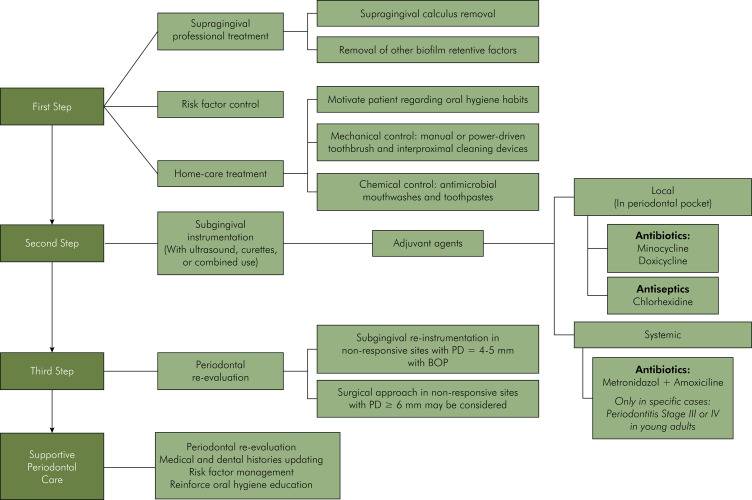
Strategies for managing periodontitis.
- **Patient involvement:** This stresses the necessity of patient engagement in biofilm control by means of home-care and professional interventions for long-term periodontal health.
- **Tailored SPC programs:** The need for personalized long-term SPC programs that integrate oral and systemic health, focusing on identifying and addressing factors affecting patient adherence, is underscored.
- **Education and clinical practices:** The consensus called for updates in dental education and clinical practices in LACC, advocating for the adoption of evidence-based, cost-effective, and feasible periodontal care strategies.
- **Public health policies:** A strong advocacy for comprehensive public health policies is made, emphasizing preventive measures, early interventions for periodontal health, and integration of oral health within overall health and healthy lifestyles.

### Research gaps and future needs

- **Patient education and motivation strategies**: There is a critical gap in understanding the best patient education and motivation strategies for effective oral hygiene maintenance in LACC. Current research indicates a need for more innovative approaches beyond traditional methods. Future research should explore interdisciplinary strategies, incorporating insights from psychology, sociology, and education, to develop more effective patient communication and educational models tailored for LACC. This could include digital health interventions, community-based programs, and culturally tailored educational materials that resonate with diverse populations.
- **Long-term outcomes of periodontal treatment in LACC**: There is also a significant lack of data regarding the long-term outcomes of various periodontal treatments, especially in diverse socioeconomic and cultural settings. This gap hinders the development of tailored treatment protocols and public health policies. Future research should focus on longitudinal studies that track the efficacy of different periodontal interventions in LACC over extended periods. These studies should consider a range of variables, including patient demographics, socio-economic status, access to healthcare, and cultural attitudes toward oral health.
- **Socio-economic disparities in LACC periodontal healthcare**: Lastly, there’s an urgent need to address the socio-economic disparities that affect periodontal healthcare and its outcomes in LACC. Research should explore how these disparities influence access to and the efficacy of periodontal care. This includes understanding barriers to accessing care, such as cost, availability of services, and patient awareness, and developing strategies to overcome these challenges.

### Recommendations

- **Implement comprehensive care**: Adopt a holistic approach to periodontal treatment, tailored to each patient, integrating individual risk factor management with non-surgical and surgical treatments - the latter as required, and ongoing SPC.
- **Enhance patient involvement**: Foster a deeper engagement of patients in their periodontal treatment, underscoring the essential role of managing biofilm effectively and controlling risk factors. This should involve a synergistic approach that combines home-care practices with professional dental interventions.
- **Personalize SPC programs**: Develop tailored, long-term SPC programs that integrate oral and systemic health, focusing on identifying and addressing factors that affect patient adherence.
- **Revamp education and clinical practices**: Call for updates in dental education and clinical practice in LACC to reflect the region’s specific needs and realities. This includes adopting evidence-based, cost-effective, and feasible periodontal care strategies.
- **Enhance public health policies**: Strongly advocate for developing and enhancing comprehensive public health policies. These policies should be broad-ranging and inclusive, focusing on preventive measures and early interventions for periodontal health and integrating oral health within the broader context of overall health and healthy lifestyles.
